# Machine learning methods can replace 3D profile method in classification of amyloidogenic hexapeptides

**DOI:** 10.1186/1471-2105-14-21

**Published:** 2013-01-17

**Authors:** Jerzy Stanislawski, Malgorzata Kotulska, Olgierd Unold

**Affiliations:** 1 Institute of Computer Engineering, Control and Robotics, Wroclaw University of Technology, 50-370, Wroclaw, Poland; 2 Institute of Biomedical Engineering and Instrumentation, Wroclaw University of Technology, 50-370, Wroclaw, Poland

**Keywords:** Amyloid, 3D profile, WEKA, Alternating decision tree, Neural network

## Abstract

**Background:**

Amyloids are proteins capable of forming fibrils. Many of them underlie serious diseases, like Alzheimer disease. The number of amyloid-associated diseases is constantly increasing. Recent studies indicate that amyloidogenic properties can be associated with short segments of aminoacids, which transform the structure when exposed. A few hundreds of such peptides have been experimentally found. Experimental testing of all possible aminoacid combinations is currently not feasible. Instead, they can be predicted by computational methods. 3D profile is a physicochemical-based method that has generated the most numerous dataset - ZipperDB. However, it is computationally very demanding. Here, we show that dataset generation can be accelerated. Two methods to increase the classification efficiency of amyloidogenic candidates are presented and tested: simplified 3D profile generation and machine learning methods.

**Results:**

We generated a new dataset of hexapeptides, using more economical 3D profile algorithm, which showed very good classification overlap with ZipperDB (93.5%). The new part of our dataset contains 1779 segments, with 204 classified as amyloidogenic. The dataset of 6-residue sequences with their binary classification, based on the energy of the segment, was applied for training machine learning methods. A separate set of sequences from ZipperDB was used as a test set. The most effective methods were Alternating Decision Tree and Multilayer Perceptron. Both methods obtained area under ROC curve of 0.96, accuracy 91%, true positive rate ca. 78%, and true negative rate 95%. A few other machine learning methods also achieved a good performance. The computational time was reduced from 18-20 CPU-hours (full 3D profile) to 0.5 CPU-hours (simplified 3D profile) to seconds (machine learning).

**Conclusions:**

We showed that the simplified profile generation method does not introduce an error with regard to the original method, while increasing the computational efficiency. Our new dataset proved representative enough to use simple statistical methods for testing the amylogenicity based only on six letter sequences. Statistical machine learning methods such as Alternating Decision Tree and Multilayer Perceptron can replace the energy based classifier, with advantage of very significantly reduced computational time and simplicity to perform the analysis. Additionally, a decision tree provides a set of very easily interpretable rules.

## Background

Amyloids are proteins that can form fibrils - highly ordered aggregates of a characteristic zipper structure [1-4]. Majority of these proteins natively have a completely different functional structure in their physiological state, although functional amyloids also exist [5,6]. A hypothesis holds that *in vivo* amyloidogenic regions are usually capped by gatekeeper aminoacids, like prolines and glycines, which prevent aggregation, and may have a high affinity to chaperone proteins [7]. Very often amyloids lead to serious diseases, like Alzheimer disease (amyloid-β, tau), Parkinson disease (α-synuclein), type 2 diabetes (amylin), Creutzfeldt-Jakob disease (prion protein), Huntington disease (huntington), amyotrophic lateral sclerosis (SOD1), etc. (for a review see e.g. [5]). The number of diseases that turn out amyloid-associated is constantly increasing. It is believed that their toxicity is related to insertion of non-mature aggregates into plasma membranes as non-selective ion channels.

Recently, it was discovered that amyloidogenic properties can be due to short segments of aminoacids in a protein sequence (*hot spots*), which can transform the structure when non-burried [8]. It was proposed that hexapeptides can sufficiently represent such *hot-spots*, although they may vary between 4–10 aminoacids. A few hundreds of such peptides have been experimentally found, however testing all combinations is not possible. Instead, they can be predicted by computational methods.

Several physico-chemical methods have been proposed to predict amylogenicity of a peptide, e.g. Tango [9], ZipperDB [10,11], Pasta [12], AggreScan [13], PreAmyl [14], Zyggregator [15], CamFold [16], NetCSSP [17], FoldAmyloid [18], AmyloidMutant [19,20], BetaScan [21], and consensus AmylPred [22]. The majority of these methods predict probability of a sequence to form β-aggregates. As it turned out, such an approach was not always successful. Although β-aggregation is related to amyloidosis, structural and biophysical properties are different [7,9]. β-aggregation is quite common in highly concentrated proteins, which do not form fibers. On the other hand, certain amyloids, like prions, are poorly predicted by tools dedicated to β-aggregates.

Methods like 3D profile, applied in ZipperDB or AmyloidMutant, which take into account more specific structural features of amyloids - resembling a steric zipper [4] - work better in such cases. Also statistical elements seem to help in the classification, as shown in Waltz [23] using Position Specific Scoring Matrices (PSSM), or Bayesian classifier and weighted decision tree applied to long sequences of bacterial antibodies [24].

Experimental datasets, upon which new classification methods could be built, are still very limited. Those sequences that show amyloid propensity are rarely well characterized. For the majority of them, it is not known which segment is responsible for their amylogenicity and few of them have an experimental structure of high resolution [4]. The biggest database of potential hexapeptides, generated with the 3D profile method, comes from the ZipperDB. The classical 3D profile method applies over 2.5 thousand scaffolds resembling a steric zipper structure, on which tested hexapeptides are threaded, and their minimal energy is calculated. If the minimal energy of one chain is below a threshold value, which could be obtained from experimental dataset of hexapeptides, then the hexapeptide is classified as amyloidogenic. The method is reasonable and quite accurate - the authors of Waltz tested it on the independent dataset from prion protein sup35, which was experimentally derived. They reported that the 3D profile method showed accuracy of 0.8, with sensitivity of 0.67 and specificity of 0.84 [23]. The database in ZipperDB, which is freely available on-line [25], is constantly growing. Currently it covers all ORFs from 3 genomes: *H. sapiens*, *S. cervisiae*, and *E. coli*, with 50% redundancy. Interestingly, the database shows *hot spots* in a majority of proteins. It does not mean that they can easily turn into amyloids in the physiological conditions but it shows new interesting aspects of this topic. Unfortunately, the 3D profile method is very computationally expensive and not very simple to use.

In this paper, we propose two methods to extend the ZipperDB dataset, classifying hexapeptide candidates at lower computational cost. One of the methods is closely related to the original idea of ZipperDB, only reducing the number of profiles. The other one, which introduces the main increase of the efficiency, uses a completely different statistical approach - machine learning. Both methods are tested versus original ZipperDB database classification.

## Results and discussion

### Dataset

We generated a new dataset of 4481 hexapeptdes, which was later used for training machine learning methods (see Additional file 1, *trainset(+)* and *trainset(-)*), using our version of 3D profile method with very significantly reduced number of profiles (see Methods - Dataset), and the method of exact energy calculation proposed in ZipperDB 2006 [10]. The dataset contains 825 positively and 3656 negatively classified segments. Part of our dataset (2702 hexapeptides, see Additional file 1) is also available in ZipperDB 2010 (as of February 2012), which uses the simplified "triplet" method of calculating the energy and fuzzy logic. Energies of these hexapeptides, obtained from our study and from ZipperDB, were compared. Based on the energy criterion, 93.5% of the segments were identically classified with regard to their amyloid properties (Additional file 1). In this set, 622 (23%) hexapeptides were classified by our energy value as amyloidogenic; ZipperDB classified 612 (22.6%) as amyloidogenic. Differently classified hexapeptides often had energies close to the classification threshold of −23 kcal/mol. The mean absolute value of the energy difference, between these two sources, was 1 kcal/mol; 90% of the difference was below 1.4 kcal/mol. Energy values could differ because of limited number of threading profiles, as well as randomization element in Rosetta Design. Moreover, computationally faster but simplified triplet method, used in ZipperDB 2010, also affected the results. As stated by the ZipperDB authors, who tested it on segments of *E. coli ORFs*, an average error introduced by the triplet method was of 4 kcal/mol (90% of the difference) with the tendency to overestimate amylogenicity [11].

Our study shows that the results from our threading method are very comparable with those from ZipperDB 2010. In the new part of our dataset (1779 segments), 204 (11%) of the hexapeptides were classified as amyloidogenic (Additional file 1). Also position dependant frequencies of each aminoacid are similar in our set and the set from ZipperDB (see Methods – Dataset and Additional file 2).

### Machine learning methods

Our dataset, with a binary classification of sequence amyloid propensities based on their calculated energies, was applied for training machine learning methods [26], provided by WEKA [27]. Our objective was testing a potential for sequence based machine learning methods, which could be very significantly faster than threading and energy calculation. From a hundred of different machine learning methods, pre-selection was carried out (see Methods). Special consideration was given to methods with the highest potential for biochemical interpretation. From the preliminary tests, using cross-validation on the training set and selected efficiency measures, ten methods gave promising results: Alternating Decision Tree (ADTree) [28], Best-First Tree (BFTree) [29], Functional Tree (FT) [30], a clone of the Repeated Incremental Pruning to Produce Error Reduction (JRip) [31], a PART decision list (PART) [32], Ripple Down Rule (Ridor) [33], Support Vector Machine (SVM) method, implemented in WEKA with Sequential Minimal Optimization (SMO) algorithm for training a support vector classifier using polynomial or RBF kernels [34], MultiLayer Perceptron (MLP) [35], Naive Bayes [36], and Random Forest (RF) [37].

**Figure 1:**
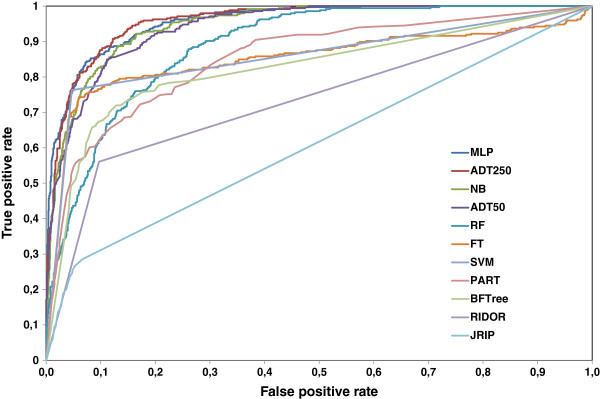
**A plot of ROC curves of all methods.** A plot of ROC curve for all the methods. Among all methods, MultiLayer Perceptron and Alternating Decision Tree with 250 boosting iterations cover the maximum area under the curve (i.e. 0.96), closely followed by Naive Bayes (AUC of 0.95) and Alternating Decision Tree with 50 boosting iterations (AUC of 0.94). In Table 1 all the corresponding AUC values are reported.

The final results of these 10 methods, using a separate test set (see Methods – Database) are shown in Table 1. The parameters of the methods (True Positive Rate - TPR, where amyloidogenic is regarded as “positive”, True Negative Rate - TNR, Area Under ROC Curve - AUC) were optimized. Top methods were selected according to their AUC ROC (Figure 1).

**Table 1:** Machine learning performance

**Method**	**TPR**	**TNR**	**Acc**	**AUC**
**MLP**	0.78	0.95	0.91	0.96
**ADTree 250**	0.78	0.95	0.91	0.96
**Naive Bayes**	0.53	0.98	0.88	0.95
**ADTree 50**	0.64	0.96	0.89	0.94
RF	0.26	0.98	0.82	0.89
FT	0.73	0.94	0.90	0.85
SVM	0.76	0.95	0.91	0.86
Part	0.56	0.94	0.86	0.85
BFTree	0.67	0.91	0.86	0.82
Ridor	0.56	0.90	0.83	0.73
Jrip	0.29	0.93	0.79	0.61

The performance evaluation of the machine learning methods. The results are ordered by decreasing AUC.

The tests showed that some of the standard WEKA methods can be very successfully used for classification of amyloidogenic segments, compatible with 3D profile method. In the best methods, Acc was typically close to 90%. The most effective methods from WEKA were MLP, ADTree, and Naive Bayes (results in Table 1, model details in Additional file 3).

**Figure 2:**
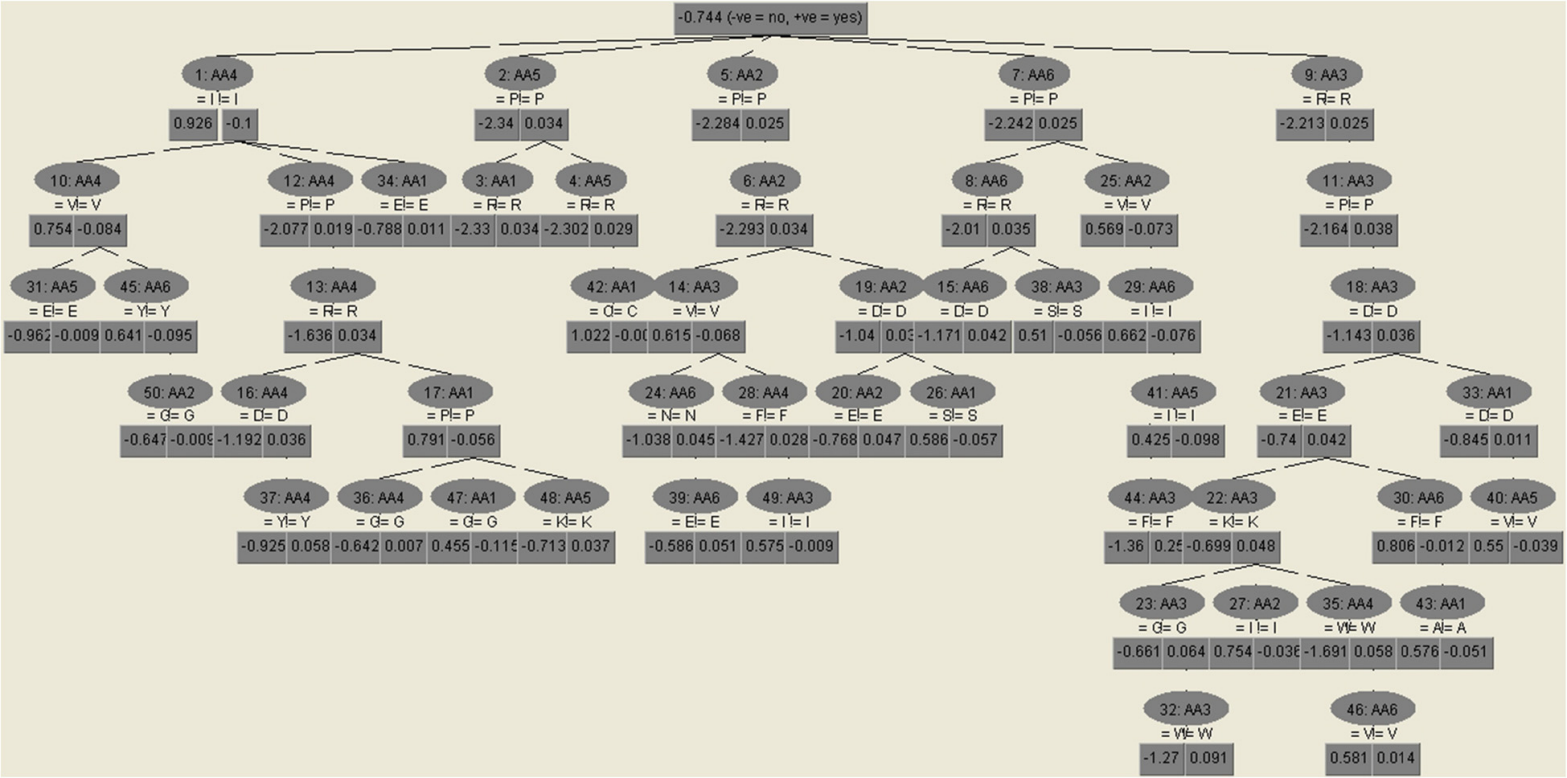
**ADTree with 50 rules.** In notation of the rules, *n*: AA *j*, *n* indicates the rule number (ordered by their significance), *j* denotes the aminoacid position. Below the rule label, the aminoacid occurrence (or absence marked by “!”) are valued by numbers. Negative numbers denote low aminoacid occurrence. A detailed explanation on how to read the Alternating Decision Tree is given in the main text.

The best ADTree, with 250 rules, achieved AUC=0.96, which is close to maximum AUC=1, characteristic of an ideal classifier, TPR=0.78, TNR= 0.95, and Acc= 0.91. Identical results were achieved with MLP classifier. We have also tested if removal of hexapaptides that overlap experimental datasets and introduce a bias in sequences, coming from the highly redundant AmylHex dataset, influence the result of our classifiers. The ADTree 250 trained on the reduced training set, deprived of hexapeptides overlapping AmylHex and Waltz, showed higher efficiency – it obtained AUC=0.98, TPR=0.81, TNR=0.96, and Acc=0.94. The rules (trained on the full set) concerning each hexapeptide position are presented and compared in Additional file 4. There are some differences between trees built on the full and reduced training sets. For example, the tree from the full training set favored valine at position 3 (rule 14 with factor 0.6, Additional file 4), overrepresented in AmylHex, while the tree built on the reduced dataset shows valine only in rule 47 (less significant), and with factor 0.45. The trees with a lower number of rules, which are easier for interpretation, are also good classifiers (see ADTree with 50 rules in Figure 2). The top rules for this tree are fairly compatible with the PSSM underlying Waltz method reported in [23], and they indicate, for example, that isoleucine (I) is highly expected at position 4 (see ADTree with 50 rules in Figure 2, rule 1: AA4=I with the factor of 0.926), while proline (P) and arginine (R) are not welcome (Figure 2, ADTree with 50 rules, rule 12: AA4=P with the factor of −2.077, and rule 13:AA4=R with the factor of −1.636, respectively).

Efficiency of the MLP method was not sensitive to the presence of the redundant hexapeptides in the training set (results in Additional file 3). Naive Bayes improved its efficiency when trained on the reduced set (AUC=0.97, TPR=0.54, TNR=0.99, Acc=0.91).

A representative estimate of WEKA methods performance, which was independent of class distribution and the specifics of the training data set (see Methods) is presented in Table 2, showing the number of wins, draws and losses when all methods are compared to each other for AUC. The corrected paired t-test showed that ADTree and MLP had statistically significantly higher AUCs than other methods, at the 5% of the significance level.

**Table 2:** Statistical evaluation

**Method**	**Wins**	**Draws**	**Losses**
MLP	10	0	0
ADTree 250	8	1	1
Naive Bayes	6	3	1
ADTree 50	6	2	2
FT	3	5	2
SVM	3	3	4
PART	3	3	4
RF	3	3	4
Ridor	1	1	8
BFTree	1	1	8
JRip	0	0	10

Summary of wins, draws and losses of each method confronted with others (with regard to their AUCs).

### Compatibility of the energy based classification with FoldAmyloid and Waltz

To test to what extent the 3D profile method overlaps with other state of the art methods, i.e. how universal its extended datasets could be, we performed the amylogenicity prediction with other tools: FoldAmyloid and Waltz, using different classification methods. FoldAmyloid is based on the packing density [23] and Waltz is based on PSSMs primarily derived from a dataset classified by physicochemical modeling [9]. The whole set of 4481 hexapeptides was tested, using all FoldAmyloid options and Waltz optimizations for overall performance and sensitivity (Additional file 5). The results are summarized in Table 3.

**Table 3:** ZipperDB versus other methods

**Method**	**Overlap**	**Positive**	**Negative**
1. FoldAmyloid *Contacts*	0.77	0.36	0.86
2. FoldAmyloid *Bone-Bone Donors*	0.79	0.39	0.88
3. FoldAmyloid *Bone-Bone Acceptors*	0.78	0.39	0.86
4. FoldAmyloid *Contacts+Donors*	0.78	0.38	0.87
**5. FoldAmyloid *Contacts+Donors+Acceptors***	**0.77**	**0.43**	**0.84**
6. Waltz *Best Performance*	0.81	0.11	0.97
7. Waltz *High Sensitivity*	0.80	0.30	0.91
**CONSENSUS** 3D profile + (5) + (7)	0.69	0.21	0.65

Compatibility of the 3D profile classification with FoldAmyloid and Waltz.

The overall classification overlap was similar in all methods; typically 80% of the hexapeptides were classified identically, using 3D profile and FoldAmyloid or Waltz. On the other hand, 84-88% of hexapeptides, classified by 3D profile as non-amyloidogenic, were also negatively classified by FoldAmyloid; Waltz overlap of negatives was 91-97%. However, classification of the positive hexapeptides was less compatible - ranging from 11% in Waltz *best performance* method to 43% in FoldAmyloid t*riple hybrid* method. This result shows that classification of positive instances is more challenging and should become the target. Less numerous positive datasets of experimental data, on which all classification methods were previously trained, could contribute to this situation. Also in our dataset, only 18.4% of hexapeptides were regarded as positive. Importantly, recognition of non-amyloid segments in the optimal method overlapped in 84%. This means that negative peptides can be eliminated efficiently and consistently between different methods. We have also tested the consensus between 3D profile, FoldAmyloid triple hybrid and Waltz high sensitivity. It turned out that the overlap was 69%, in which positive rate was 21% and negative 65%.

## Conclusions

Extending of the hexapeptide dataset, with computationally effective methods, could help in predicting amyloidogenic regions without laboratory experiments, which are currently not possible on all sequence combinations. We proposed an optimization to the classical 3D profile method, and using only 54 arbitrarily selected profiles we generated the new dataset of hexapeptides classified with regard to their amylogenicity. Energies of our segments showed very good overlap with the segments currently available in ZipperDB, which used the simplified Triplet method to calculate the energy. The new part of our dataset contains 1779 segments that have not been previously considered, with 204 segments classified by energy value as amyloidogenic.

We also performed the amylogenicity prediction on our dataset, using different classification methods - FoldAmyloid and Waltz. The best result was obtained with FoldAmyloid triple hybrid method, which overlapped the 3D profile classification in 77% (total), 43% (amyloidogenic), and 84% (non-amyloidogenic). It showed that different methods are quite compatible in the elimination process, and in this respect datasets generated with the 3D profile methods are universal.

To test whether statistical approach, trained on our dataset, could replace the energy based classifier, we used machine learning methods implemented in WEKA. Our dataset of 6-residue sequences, with a binary classification of their amyloidogenic propensities based on the calculated energy, was applied for training. From all available WEKA methods, we selected those giving the best results and tested with a separate test set, obtained from ZipperDB. Our study showed that some of the methods could be very successfully used for classification of amyloidogenic segments, compatible to the 3D profile method. The most effective methods in WEKA, in terms of AUC ROC, were Alternating Decision Tree and a Neural Network of a Multilayer Perceptron architecture, both with AUC=0.96. The ADTree efficiency could be increased to AUC=0.98 when highly redundant set of experimental hexapeptides was removed from the training set. The performance was then very close to an ideal classifier, for which AUC=1. A great advantage of ADTree method is a set of very easily interpretable rules. Part of the rules were fairly compatible with the PSSM underlying another classification method -Waltz, which was based on different data All those methods could classify almost 80% of positive and 95% of negative hexapeptides identically as the 3D profile method.

Such a good result of classification, based only on aminoacid sequence and its binary classification, is very interesting. It shows a good correlation between classification with the laborious 3D profile method ­ using the minimal chain energy from numerous putative structures, and purely statistical machine learning methods - using just 6 letters and the binary classification. This is possible only if a strong statistical pattern exists in the amyloid sequences recognized by 3D profile. Our results also prove that our new dataset is representative enough for training machine learning methods, in order to obtain amylogenicity of new segments only based on their six letter sequences, with no need to carry out threading procedure and energy evaluation.

The main advantage of the machine learning approach, presented in this paper, is very significantly reduced computational time. Instead of 18–20 CPU-hours with the full 3D profile method or 0.5 CPU-hours with the simplified 3D profile, the classification can take below 1 CPU-minute with a very good overlap of the results. Such a reduction of the computational time is crucial when large amount of hexapeptides should be classified. Additionally, the machine learning enhances the simplicity to perform the analysis.

## Methods

### Database

As a reference dataset of 6-residue sequences, also applied to test our machine learning results, we used the first set published in ZipperDB as of 2010 (Additional file 1: testset(+) and testset(−)) [11].

The set used for training machine learning methods was obtained from non-redundant protein sequences of UniProt [38], cut into 6-residue windows by shifting of 1 position along the full sequence. The hexapeptides were then divided into amylo-positive and amylo-negative candidates with our simplified 3D profile method. To increase chances of finding amyloid segments, UniProt entries containing the keyword “amyloid” and proteins from AmyPDB database [39,40] were selected for the procedure (both accessed in September 2010). The sequences were first cut into strings not exceeding 80 aminoacids each, and excessive redundancy was reduced at the level of 90%, with CD-HIT (Cluster Database at High Identity with Tolerance) [41,42]. Next, the remaining sequences were cut into hexapeptides with a window of length 6, shifted of 1 position in each move. The set was finally enlarged with 266 hexapeptides studied experimentally: AmylHex dataset [10] and Waltz [23].

The full dataset do not show position dependence of aminoacids, with statistics close to the frequencies of aminoacids in all UniProt [43]. Even closer statistics were obtained for the dataset not enriched with the hexapeptides from AmylHex and Waltz. The statistics of the test set is also close to the same characteristics, although some numbers slightly differ (e.g. prolines are excluded in the test set). Standard deviations are negligible in both datasets. The statistics of datasets, in the form of tables and logos (prepared with WebLogo [44]), are presented in Additional file 2.

### Threading and energy calculation

First, all cysteines were replaced by serines to avoid disulfide bridges as in [10]. Similarly to 3D profile method [10], a fibril-forming peptide NNQQNY, from sup35 prion protein of Saccharomyces, was used as a scaffold in the threading method. Each hexapeptide from the set was threaded on the scaffold; 5 identical copies of the hexapeptide formed one of two identical β-sheets. In the final structure, one of the β-sheets was shifted relative to the other one. In our implementation, which differs from 3D profile method, the movement was exercised in two planes: along the chains of 0-8 Å with the step of 1 Å, and across the sheets fixing the distance between them to 6–11 Å with the step of 1 Å. We did not use the third direction, which was implemented in [10]. Finally, we obtained 54 profiles, instead of 2,511 in full method [10]; no fuzzy logic was used to reduce the number of profiles as in [10]. Then, the energies of 54 profiles were calculated. For each segment from the dataset the energy was obtained with Rosetta Design program [45,46], which added the side chains to the backbones, applied a random component of the simulated annealing to relax the structure, and calculated the energy of infinite periodic system (for more details of the energy calculation procedure see [45]). As an optimal configuration, for each hexapeptide a structure with minimal energy was selected from the set of profiles. Similarly as in [10], the threshold of −23 kcal/mol was assumed to classify amylogenicity of a segment. Positive instances contained at least one chain whose energy was not greater than the threshold.

The full original 3D profile method, evaluating a single 6-residue segment, required 18–20 CPU-hours (2.5 GHz AMD Opteron, Phenom or Intel Xeon CPU), while 2–2.5 CPU hours were needed with the triplet method and fuzzy logic selecting 80–100 templates [11], supplement. Our method applies the original method, only reducing the number of templates 46 times (from 2 500 to 54). The energy calculation would take 0.5 CPU-hour for each hexapeptide, with the same computer.

### Machine learning

The classifiers were trained on all 4481 hexapeptides from our dataset obtained by simplified 3D profile method (Additional file 1). Prediction methods were provided by WEKA 3.6.6 (Waikato Environment for Knowledge Analysis) [27],which includes a hundred of different classifiers. Pre-selection, to find the most effective methods, was carried out with default WEKA sets of parameters and 10-fold cross-validation on the training set. Next, we chose 10 most promising methods from WEKA suite. Finally, we used the following methods with the optimized parameters:

ADTree (numOfBoostingInterations=250 or 50, randomSeed=1),

BFTree (minNumObj=4, numFoldsPruning=4, seed=2, pruningStrategy=un-pruned),

FT (minNumInstances=26, numBoostingIterations=86),

RF (maxDepth=unlimited, numFeatures=log2(7)+1, numTrees=200, seed=1),

JRip (folds=4, minNo=1, optimizations=7, seed=4),

MLP (hiddenLayers=1 with 60 nodes, learningRate=0.1, Momentum=0.2, seed=0, trainingTime=500),

PART (confidenceFactor=0.3, mniNumObj=1, numFold=3, seed=1),

Ridor (folds=3, minNo=6, seed=1, shuffle=7),

SVM (c=2.0, kernel=linear)

Naive Bayes (no parameters).

Parameters not specified have their values set to default.

Out of the above mentioned methods, ADTree algorithm has several advantages over other machine learning methods, such as MLP or SVM, including easy interpretation of the results. An alternating decision tree is in fact a graphical representation of a collection of user interpretable rules. Each tree consists of decision and prediction nodes. The prediction node contains a single number, whereas decision node defines a predicate conditions. To classify an instance, the ADTree follows all paths for which all decision nodes are true. The final classification score is gained by summing all the prediction nodes through which the instance it passes.

### Prediction accuracy assessment

The binary test set included 346 positive and 1240 negative hexapeptides from ZipperDB, as of May 2010 (Additional file 1). The classification results were evaluated based on typical measures: Sensitivity (called True Positive Rate - TPR), Specificity (True Negative Rate - TNR), Accuracy (Acc). These criteria widely used to evaluate the performance of prediction models, and defined as below:

$$\begin{array}{c} \mathrm{TPR}=\mathrm{TP}/\left(\mathrm{TP}+\mathrm{FN}\right),\\ \mathrm{TNR}=\mathrm{TN}/\left(\mathrm{TN}+\mathrm{FP}\right),\\ \mathrm{Accuracy}=\left(\mathrm{TP}+\mathrm{TN}\right)/\left(\mathrm{TP}+\mathrm{TN}+\mathrm{FP}+\mathrm{FN}\right), \end{array}$$

where TP, FP, FN and TN represent the numbers of true positives, false positives, false negatives and true negatives respectively. The overall quality of a classifier can be evaluated with area under ROC curve (AUC) [47]. The value of the AUC score ranges from zero to one, with a score of 0.5 corresponding to random guess and a score of 1.0 indicating perfect separation. This estimator evaluates the method in separating amyloids from non-amyloids. In particular, The AUC is well received in the imbalanced dataset community and it is becoming the standard evaluation method.

### Statistical validation

In order to obtain a representative estimate of WEKA methods performance, which is independent of class distribution and the specifics of the training data set, we performed an experiment with 10 train and test runs. The data used for training and testing - a dataset of 4481 hexapeptides - was randomly divided into 66% and 34%, respectively. The results were analysed statistically using a corrected paired samples t-test [46] where we computed p-values at the 5% significance level, comparing every method with every other method for their AUC. This is a parametric procedure used to determine whether there is a significant difference between the average values of the same performance measure for two different methods. The test assumes that the paired differences are independent and identically normally distributed. Although the measurements themselves may not be normally distributed, the pair wise differences often are.

### Validation with other classification methods

Two different state of the art methods were used to test the inter-compatibility of the energy based method: FoldAmyloid [18,48] and Waltz [23,49] (as of March 2012). We performed the analysis on the whole set of 4481 hexapeptides, using our scripts in Python programming language and spynner - open source web browsing module for communication with both services. All standard FoldAmyloid methods were applied: contacts, bone-bone donors, bone-bone acceptors, hybrid (contacts + donors), and triple hybrid (contacts + donors + acceptors). Waltz was run with its standard optimizations for overall performance and sensitivity.

## Competing interests

The authors declare that they have no competing interests.

## Authors’ contributions

JS proposed the simplified 3D profile method, generated the dataset and did preliminary tests on the machine learning methods. MK validated the dataset with ZipperDB, ran and analyzed machine learning, FoldAmyloid and Waltz methods, and drafted the manuscript. OU ran and analyzed machine learning methods, performed the statistical analysis, and participated in writing the manuscript. MK and OU designed and supervised the study. All authors read and approved the final manuscript.

## Supplementary Material

Additional file 1 Dataset of hexapeptides with calculated energies and amylogenic classification. Click here for file

Additional file 2 Position specific aminoacid frequencies of the training and test datasets. Click here for file

Additional file 3 Detailed results of the best machine learning methods trained on the full and reduced training sets. Click here for file

Additional file 4 **Rules of ADTree 250 methods for full and reduced training sets.** Columns contain the rules corresponding to each position in amyloidogenic hexapeptides in decreasing order of their importance. Yellow cells denote positive rules, purple – negative rules. Click here for file

Additional file 5 **Amylogenic classification of our dataset obtained with reduced 3D profile (additional file 1: trainset(+) and trainset(-)) with different methods: 3D profile, FoldAmyloid and Waltz.** The file includes the spreadsheets labeled according to the name of the external method. Click here for file
