# Supplementary material for: Machine learning methods can replace 3D profile method in classification of amyloidogenic hexapeptides
Source: BMC Bioinformatics. 2013 Jan 17;14:21. doi: 10.1186/1471-2105-14-21 (PMC3566972; doi:10.1186/1471-2105-14-21)
Supplement: Additional file 2 — Position specific aminoacid frequencies of the training and test datasets. [file 1471-2105-14-21-S2.pdf]

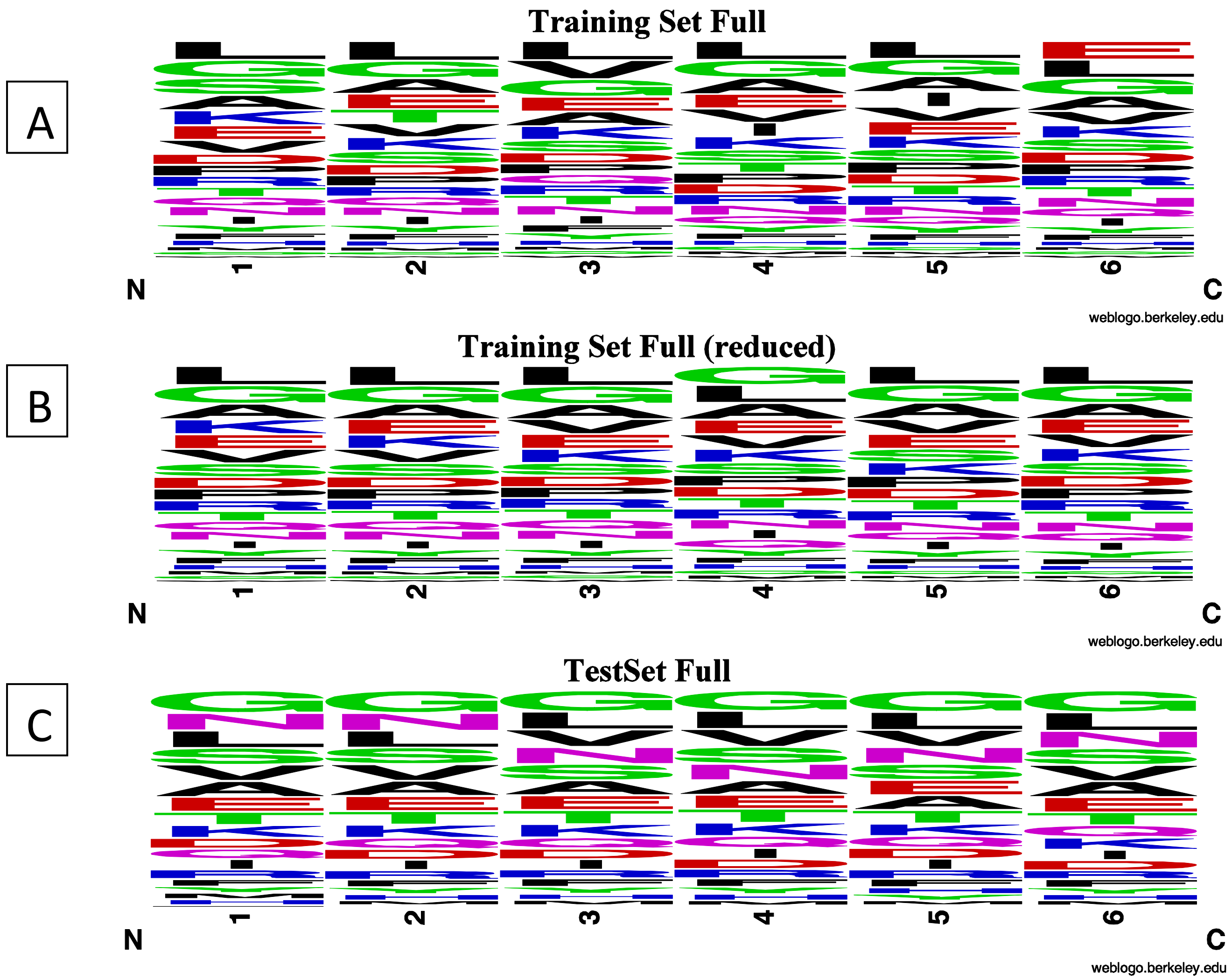

Fig.1_File2. Frequencies of aminoacids at each position of 6-residue sequences

position	1	2	3	4	5	6	stdev	
K	1.3	1.2	1.2	1.1	1.1	1.2	0.1	
R	0.9	0.9	0.9	0.9	0.9	0.9	0.0	
D	1.0	1.0	0.9	0.9	0.9	1.0	0.0	
E	1.1	1.1	1.1	1.1	1.1	1.4	0.1	
N	1.1	1.1	1.1	1.2	1.1	1.2	0.0	
Q	1.1	1.2	1.2	1.1	1.1	1.2	0.1	
P	1.1	1.1	1.0	1.1	1.1	1.1	0.0	
H	1.3	1.3	1.2	1.2	1.1	1.1	0.1	
M	0.9	0.9	0.9	0.8	0.8	0.8	0.1	
C	1.5	1.4	1.6	1.8	1.7	1.6	0.1	
S	1.2	0.9	0.9	0.8	0.9	0.9	0.1	
T	0.8	1.2	0.8	0.9	0.9	0.9	0.1	
F	0.8	0.9	0.9	0.8	0.8	0.8	0.0	
W	1.1	1.1	1.4	1.3	1.2	1.3	0.1	
Y	1.2	1.2	1.1	1.2	1.3	1.1	0.1	
V	0.9	0.9	1.3	1.0	1.0	1.0	0.1	
L	0.9	0.9	0.9	0.9	0.8	0.9	0.0	
I	0.7	0.7	0.7	1.1	1.2	0.7	0.2	
G	1.2	1.1	1.2	1.2	1.1	1.2	0.0	mean std
A	0.8	0.8	0.8	0.9	0.8	0.8	0.0	0.1
stdev	0.2	0.2	0.2	0.2	0.2	0.2		
						0.2		

position	1	2	3	4	5	6	stdev	
K	1.1	1.1	1.2	1.1	1.1	1.0	0.0	
R	0.8	0.8	0.8	0.8	0.8	0.7	0.0	
D	0.9	0.9	0.9	0.9	0.9	0.9	0.0	
E	1.1	1.1	1.1	1.0	1.1	1.1	0.0	
N	2.0	2.0	1.9	1.8	1.9	2.0	0.1	
Q	1.2	1.3	1.3	1.4	1.4	1.4	0.1	
P	0.0	0.0	0.0	0.0	0.0	0.0	0.0	
H	1.3	1.3	1.3	1.4	1.4	1.3	0.1	
M	1.2	0.9	1.0	0.9	0.9	0.9	0.1	
C	0.4	0.4	0.5	0.5	0.5	0.6	0.1	
S	1.2	1.1	1.1	1.2	1.1	1.1	0.0	
T	1.1	1.1	1.2	1.1	1.1	1.1	0.0	
F	0.8	0.8	1.0	1.0	1.0	1.0	0.1	
W	0.8	0.7	0.7	0.6	0.6	0.5	0.1	
Y	1.0	1.1	1.0	1.1	1.0	1.0	0.0	
V	1.1	1.1	1.2	1.2	1.2	1.1	0.0	
L	0.8	0.8	0.8	0.8	0.8	0.8	0.0	
I	0.8	0.8	0.8	0.8	0.8	0.8	0.0	
G	1.5	1.5	1.4	1.3	1.4	1.4	0.1	mean std
A	0.9	0.8	0.8	0.8	0.8	0.9	0.0	0.0
stdev	0.4	0.4	0.4	0.4	0.4	0.4		
						0.4		

Tab.1_File2. Frequencies of aminoacids at each position of 6-residue sequences, normalized by mean frequecies from UniProt. Training (left) and test (right) datasets do not show position dependence of aminoacids, although some frequencies of aminoacids slightly differ from those calculated by UniProt (e.g. prolines are excluded in the test set). Standard deviations are negligible.

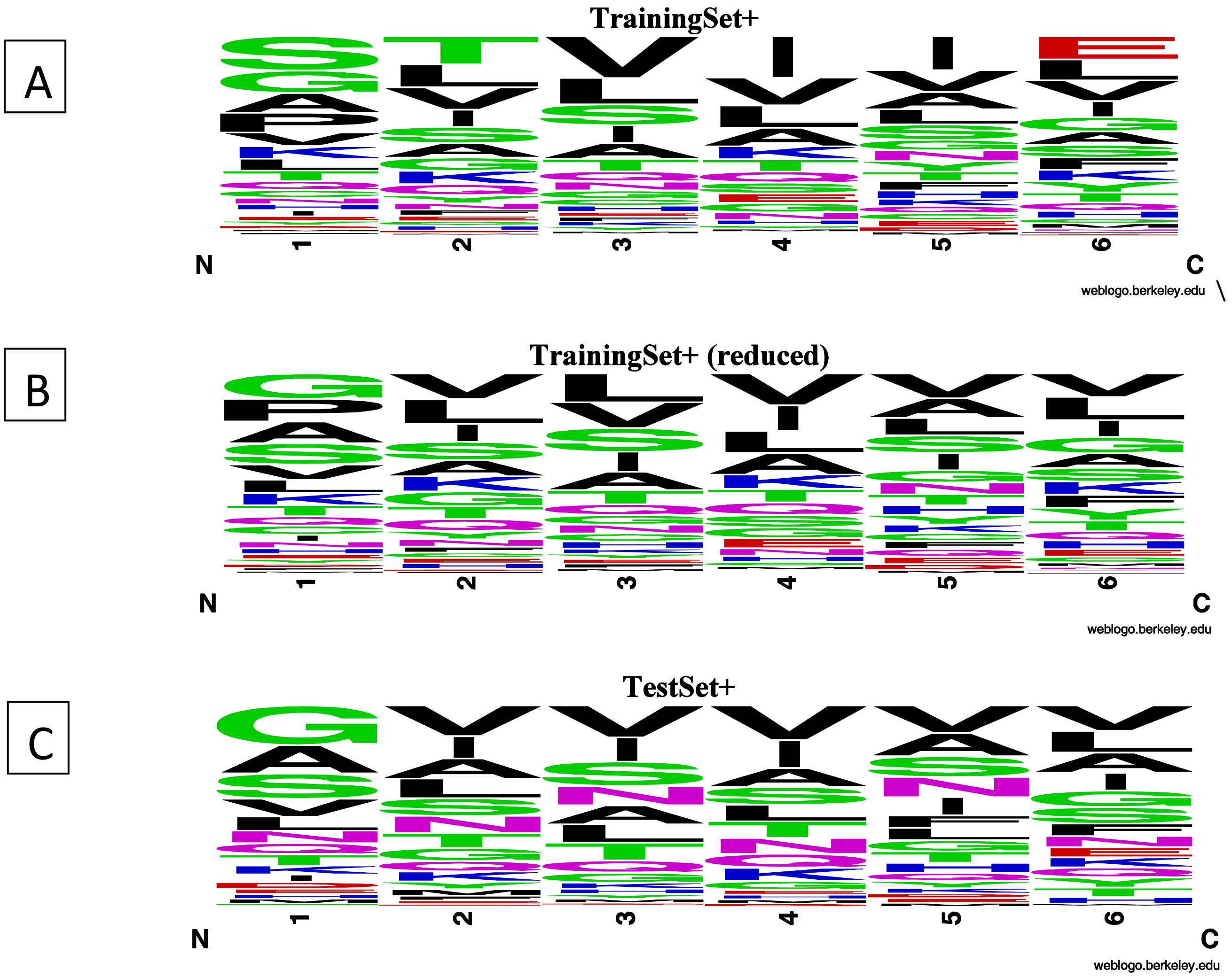

Fig.2_File2. Logo representations of aminoacid frequencies for amylo-positive computational (training, testing) and experimental datasets. Colors are the same as in Fig1_File2.

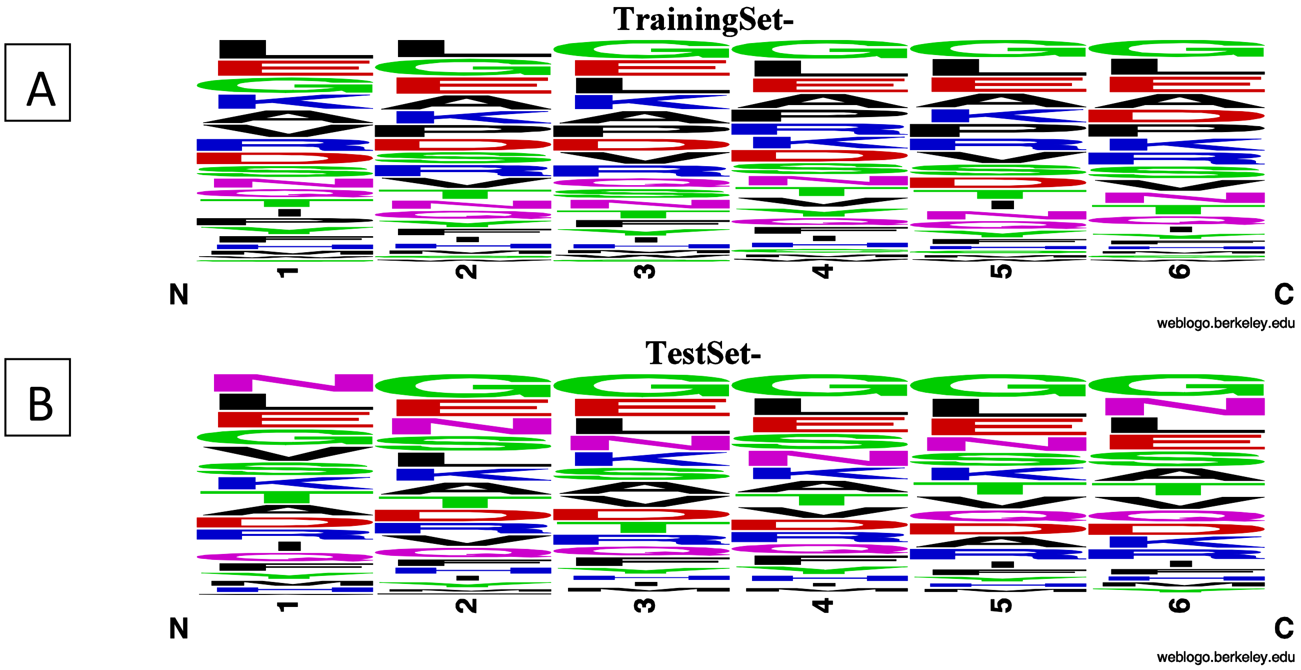

Fig.3_File2. Logo representations of aminoacid frequencies for amylo-negative computational (training, testing) .
